# Transcriptomic and epigenetic profiling of ‘diffuse midline gliomas, H3 K27M-mutant’ discriminate two subgroups based on the type of histone H3 mutated and not supratentorial or infratentorial location

**DOI:** 10.1186/s40478-018-0614-1

**Published:** 2018-11-05

**Authors:** David Castel, Cathy Philippe, Thomas Kergrohen, Martin Sill, Jane Merlevede, Emilie Barret, Stéphanie Puget, Christian Sainte-Rose, Christof M. Kramm, Chris Jones, Pascale Varlet, Stefan M. Pfister, Jacques Grill, David T. W. Jones, Marie-Anne Debily

**Affiliations:** 1 UMR8203,Vectorologie et Nouvelles Thérapies Anticancéreuses, CNRS, Gustave Roussy, Univ. Paris-Sud, Université Paris-Saclay, 94805 Villejuif, France; 2 Département de Cancérologie de l’Enfant et de l’Adolescent, Institut de Cancérologie Gustave Roussy, Université Paris-Sud, Université Paris-Saclay, 114 rue Édouard Vaillant, 94805 Villejuif Cedex, France; 3 Hopp Children’s Cancer Center at the NCT Heidelberg (KiTZ), Heidelberg, Germany; 4 Division of Pediatric Neurooncology (B062), German Cancer Research Center (DKFZ) and German Cancer Consortium (DKTK), Im Neuenheimer Feld 280, 69120 Heidelberg, Germany; 5 Department of Pediatric Neurosurgery, Hôpital Necker-Enfants Malades, Université Paris V Descartes, Sorbonne Paris Cité, Paris, France; 6 Division of Pediatric Hematology and Oncology, University Medical Center Goettingen, Goettingen, Germany; 7 Divisions of Molecular Pathology and Cancer Therapeutics, The Institute of Cancer Research, Sutton, Surrey UK; 8 Department of Neuropathology, Hôpital Sainte-Anne, Université Paris V Descartes, Sorbonne Paris Cité, Paris, France; 9 Department of Pediatric Hematology and Oncology, Heidelberg University Hospital, Heidelberg, Germany; 10 Pediatric Glioma Research Group, German Cancer Research Center (DKFZ) and German Cancer Consortium (DKTK), Im Neuenheimer Feld 280, 69120 Heidelberg, Germany; 11 Université Evry, Université Paris-Saclay, 91057 Evry Cedex, France; 12 NeuroSpin/UNATI, CEA, Université Paris-Saclay, Gif-sur-Yvette, France; 13 Univ. Evry, Université Paris-Saclay, 91057 Evry Cedex, France

**Keywords:** Pediatric high-grade glioma, Diffuse midline glioma, H3 K27M-mutant, Diffuse intrinsic pontine glioma, Epigenetics, DNA methylation profiling, Gene expression profiling, H3K27me3 landscape, Glioma stem cell

## Abstract

Diffuse midline glioma (DMG), H3 K27M-mutant, is a new entity in the updated WHO classification grouping together diffuse intrinsic pontine gliomas and infiltrating glial neoplasms of the midline harboring the same canonical mutation at the Lysine 27 of the histones H3 tail.

Two hundred and fifteen patients younger than 18 years old with centrally-reviewed pediatric high-grade gliomas (pHGG) were included in this study. Comprehensive transcriptomic (*n* = 140) and methylation (*n* = 80) profiling was performed depending on the material available, in order to assess the biological uniqueness of this new entity compared to other midline and hemispheric pHGG.

Tumor classification based on gene expression (GE) data highlighted the similarity of K27M DMG independently of their location along the midline. T-distributed Stochastic Neighbor Embedding (tSNE) analysis of methylation profiling confirms the discrimination of DMG from other well defined supratentorial tumor subgroups. Patients with diffuse intrinsic pontine gliomas (DIPG) and thalamic DMG exhibited a similarly poor prognosis (11.1 and 10.8 months median overall survival, respectively). Interestingly, H3.1-K27M and H3.3-K27M primary tumor samples could be distinguished based both on their GE and DNA methylation profiles, suggesting that they might arise from a different precursor or from a different epigenetic reorganization.

These differences in DNA methylation profiles were conserved in glioma stem-like cell culture models of DIPG which mimicked their corresponding primary tumor. ChIP-seq profiling of H3K27me3 in these models indicate that H3.3-K27M mutated DIPG stem cells exhibit higher levels of H3K27 trimethylation which are correlated with fewer genes expressed by RNAseq. When considering the global distribution of the H3K27me3 mark, we observed that intergenic regions were more trimethylated in the H3.3-K27M mutated cells compared to the H3.1-K27M mutated ones.

H3 K27M-mutant DMG represent a homogenous group of neoplasms compared to other pediatric gliomas that could be further separated based on the type of histone H3 variant mutated and their respective epigenetic landscapes. As these characteristics drive different phenotypes, these findings may have important implication for the design of future trials in these specific types of neoplasms.

**Electronic supplementary material:**

The online version of this article (10.1186/s40478-018-0614-1) contains supplementary material, which is available to authorized users.

## Introduction

Diffuse intrinsic pontine glioma and malignant midline gliomas have the worst prognosis of all types of malignant tumors in children and adolescents [3, 4, 10]. The nosological shift in the 2016 WHO classification now based on both phenotype and genotype has redefined the family tree of diffuse gliomas [17]. Glial tumors are now grouped according to their driver mutation, e.g. *IDH1* mutation, and their astrocytic or oligodendroglial phenotypes which are often associated with additional specific genetic alterations such as *ATRX* mutations or 1p/19q co-deletion, respectively. The discovery of recurrent mutations in the histone H3 genes in pediatric high-grade glioma has definitively separated these gliomas from the ones seen in adults [21, 26]. While G34R/V mutations in the *H3F3A* gene are exclusively found in the hemispheres, K27M/I mutations in several histone H3 variants genes are specific to midline tumors [23]. The 2016 release of the WHO classification has therefore created a new entity to describe these latter tumors as diffuse midline glioma, H3K27M mutant, irrespective of their specific location along the midline.

In pediatric brain tumors, location has however long been seen as a master driver of oncogenesis that could reflect their different cells of origin [8, 9]. Whether the oncogenic driver mutation is overriding location as a crucial determinant of oncogenesis is therefore to be examined since biologic identity of all these tumors would call for a common therapeutic framework. There is however no reported data showing at once a similar biology and outcome of diffuse midline gliomas (DMG) irrespective of their location in the presence of a histone H3-K27M mutation.

Moreover, we have shown two distinct forms of diffuse intrinsic pontine gliomas according to the type of histone H3 gene mutated, *H3F3A* *versus* *HIST1H3B,* with respect to differentiation markers, oncogenic programs, response to therapy and evolution [1, 2]. These mutations are mutually exclusive either because their effect is redundant [16] leading to a global loss of H3K27me3 repressive mark, or because they cannot transform the same cell, suggesting the idea of distinct cells of origin.

The purpose of this work was therefore to better characterize a large series of pediatric midline high grade gliomas from the (epi)genomic, transcriptomic and anatomic point of view in order to identify the respective influences of these parameters on their biology described by their gene expression, methylome, and clinical behaviour.

Moreover, we compared the H3-K27me3 landscape between the two main subgroups of DIPG, H3.1-K27M and H3.3-K27M, in patient deriving cellular models.

## Materials & methods

### Central pathology review

High-grade glioma cases were reviewed centrally to confirm the diagnosis according to the 2007 WHO classification and its 2016 update as previously described [10, 20].

Specific immunostainings were performed to detect nuclear expression of the trimethylation mark at position K27 of the histone 3 tail (1:1000, polyclonal rabbit antibody, Diagenode, Belgium) as well as nuclear expression of the K27M form of histone H3 (1:1000, polyclonal rabbit antibody, Millipore, CA).

### Derivation and culture of glioma stem-like cells (GSCs)

GSCs were derived from DIPG tumors at diagnosis as previously described [19]. Briefly, tumor cells were mechanically dissociated from biopsies within 24 h of surgery, and further cultured as an adherent monolayer in laminin-coated flask (Sigma) in neural stem cells medium consisting of NeuroCult NS-A proliferation medium (Stemcell technologies) supplemented with heparin (2 μg/mL, Stemcell technologies), human-basic FGF (20 ng/ml, Peprotech), human-EGF (20 ng/ml, Peprotech), PDGF-AA (10 ng/ml, Peprotech), and PDGF-BB (10 ng/ml, Perprotech). Medium was renewed every other day, and passaging performed when cells reached 80% confluence using Accutase (Thermo).

### Case selection for overall survival analysis and gene expression profiling by microarray

Frozen tissue samples were obtained from 119 pediatric patients with brain tumors of WHO grade III and IV (all locations, below 18 years old). The samples were collected at Necker Hospital (Paris, France). Complete follow-up information was available for 82.5% of patients (*n* = 99). Histone H3 gene mutational status was determined by Sanger sequencing for *H3F3A*, *HIST1H3B/C* and *HIST2H3C* [2]. The distribution of samples in the distinct genotype subgroups and location are detailed in Table 1.

**Table 1:** Contingency table of samples used for microarray gene expression profiling and overall survival analysis

Tumor location	Histone H3 mutational status				Total number of samples
	H3.3-G34R	H3.1-K27M	H3.3-K27M	H3.1 & H3.3-WT	
Cortex	6	0	0	35	41
Pons	0	13	26	6	45
Non-thalamic midline	0	0	2	12	14
Thalamic midline	0	0	12	7	19

One hundred and nineteen high-grade glioma samples were divided in four groups according to their location either in the pontine (DIPG), cortical or thalamic area of the brain as well as the non-thalamic midline, i.e. spinal cord, cerebellum or peduncle tumors classified as ‘non-thalamic midlines’

### Case and sample selections for methylation analysis

Eighty primary tumor samples were selected for methylome analysis: 22 among the DIPG patient cohort collected in Necker Hospital; 15 from the HERBY trial [10], all the remaining samples were collected by the Heidelberg group. The distribution of samples in the distinct genotype subgroups and their location are detailed in Table 2.

**Table 2:** Contingency table of samples used for methylation profiling

Tumor location	Histone H3 mutational status				*PDGFRA* subgroup	M*YCN* subgroup	Total number of samples
	H3.3-G34R	H3.1-K27M	H3.3-K27M	H3.2-K27M			
Cortex	10	0	0	0	10	10	30
Pons	0	12	19	1	0	0	32
Thalamic midline	0	1	17	0	0	0	18

Eighty high grade gliomas were analyzed by 450 k and EPIC Illumina bead arrays. Tumor location and histone H3 mutations or *PDGFRA* and *MYCN* molecular subgroups were considered for sample stratification

Gene expression profiling was also conducted by either microarray or RNA sequencing for 5 of these tumors. Eight glioma stem-like cell (GSC) cultures derived from patient biopsies at diagnosis and matching primary tumors were analyzed similarly [19].

### Methylation profiling

DNA was extracted from tumors and genome-wide DNA methylation analysis was performed using either the Illumina HumanMethylation450 BeadChip (450 k) or EPIC arrays. DNA methylation analysis was performed with custom approaches as previously described [12, 23]. DNA methylation profiles from 50 K27M pHGG were compared to defined supratentorial tumor subgroups, i.e. G34R-H3.3 mutated (*n* = 10), *MYCN* (*n* = 10) and *PDGFRA*/pedRTK1 (*n* = 10) subgroup tumors. For t-SNE analysis (t-Distributed Stochastic Neighbor Embedding, Rtsne package version 0.11), 428,230 uniquely mapping autosomal probes in common between the 450 k and EPIC arrays were used. The input for the t-SNE calculation is 1-Pearson correlation, weighted by variance. Clustering analyses were performed using the beta values of the top 10,000 most variably methylated probes by standard deviation. Methylation probes in the heatmap representation were reordered by unsupervised hierarchical clustering using Pearson correlation distance and median linkage.

### Microarray gene expression profiling

Gene expression analysis was conducted on an Agilent platform as previously described [2] but using RUV4 correction of batch effects [7] implemented in the R package ruv. GE data from DIPG were collected from one of our previous study [2] and microarray analysis was performed for 75 additional pHGG tumors located outside the brainstem. PCA, k-means and t-SNE analysis were performed using the same parameters as for RNA-seq data on the probes associated with the highest standard deviation. One hundred and twenty genes accounting for 0.79% of the entire probeset were selected.

### RNA-seq gene expression profiling

RNA-seq was performed on 21 primary tumor samples. Libraries were prepared using the TruSeq stranded mRNA sample preparation kit according to the supplier recommendations and paired-end sequencing was conducted on Illumina NextSeq500 to generate a mean of 150 million reads of 75 base pairs by sample. Trimmed reads were then mapped using tophat2 (v2.1.0) and bowtie2 (v2.2.5) first to the reference transcriptome, then to the reference genome for the remaining reads. Genes with a row sum of raw counts over the studied samples equal to or below 10 were filtered out to remove non-expressed genes. We handled outliers as default using minReplicatesForReplace = 7 in DESeq() function used to estimate size factors, dispersion and model coefficients. Distances between samples were computed by using ‘1-Pearson correlation coefficient’ as the distance measure. PCA and t-SNE analysis were performed on the 250 genes associated with the highest variance to keep the same proportion of genes selected with the microarray analysis. All samples were projected on the two first principal components computed with rlog transformation of the counts of the 120 genes with the highest standard deviation. Using Rtsne package (v 0.11), we applied t-SNE on the same data matrix with the Pearson correlation as a distance and the following parameters: theta = 0, perplexity = min(floor((ncol(rlog_VariableGenes)-1)/3), 30), check_duplicates = FALSE, pca = FALSE, max_iter = 10,000, verbose = TRUE, is_distance = TRUE.

RNA-seq was also performed on 6 distinct GSC models using TruSeq stranded total RNA sample preparation kit according to the supplier recommendations (Illumina) and then processed similarly as primary tumors.

### Histone ChIP-sequencing and data processing

ChIP-seq of H3K27me3 epigenetic modification was performed in 6 GSC models at Active Motif according to proprietary methods. The 75-nt sequence reads were generated on a Illumina NextSeq 500 platform, mapped using BWA algorithm and peak calling was performed using SICER1.1 algorithm [27] with cutoff FDR 1e-10 and gap parameter of 600 bp. False positive ChIP-seq peaks were removed as defined within the ENCODE blacklist [5]. Overlapping intervals between the different samples were merged, and the average number of normalized reads in the different samples were calculated for these 16,977 genomic intervals defined as ‘bound regions’. These bound regions were separated for further analysis in overlapping or not overlapping gene loci using Genecode annotation (gencode.v19.chr_patch_hapl_scaff_annotation.gtf). PCA for all samples were generated after scaling to unit variance using the PCA function from the FactoMineR package (v1.41) and plotted using Factoextra (v1.0.5).

Merging of the 3 biological replicates of H3.1- or H3.3-K27M subgroups was performed using bigWigMerge tool (UCSC kent utils, http://hgdownload.soe.ucsc.edu/admin/exe/macOSX.x86_64/). Heatmaps of H3K27me3 ChIPseq enrichment across genomic loci were calculated using deepTools version 1.5.11. ComputeMatrix was used with regions of either +/− 5 kb or +/− 10 kb around the center of the genomic intervals for ‘bound regions’ or TSS for differentially expressed genes, respectively. Heatmaps were plotted with or without k-means (*k* = 5) and their average profiles of ChIP-Seq enrichment in the same − 10/+ 10 kb genomic intervals were also generated for each k-means group. The bigWig files (all signal) were annotated with chipSeeker package using UCSC hg19 known gene annotation and visualizated by peakAnno and Vennpie.

### Survival curve comparisons

The distribution of overall survival (OS) was calculated according to the Kaplan-Meier method and all survival function estimate comparisons were performed in PRISM software using a log-rank test. OS was calculated from the date of histo-radiological diagnosis until death of patient from disease or last contact for patients who were still alive.

## Results

### Histone H3 K27M midline pHGG and K27M DIPG display similar gene expression profiles and survival but differ significantly from other high-grade gliomas

We conducted microarray gene expression profiling of the single center cohort from Necker Enfants Malades hospital of 119 pHGG with histone H3 genotype previously determined either by Whole Genome Sequencing [25] or targeted Sanger sequencing [2] (Table 1 and Additional file 1: Table S1). In total, 131 distinct gene expression microarrays were hybridized as 12 samples were analyzed twice to control for potential batch effects. The 12 duplicated samples were located in close proximity on PCA plot when projected on the 2 first principal components, confirming the appropriate removal of a batch effect in our dataset (*data not shown*). Then, we selected a set of genes associated with the highest standard deviation for subsequent tumor classification analysis (*n* = 120). Gene Ontology over-representation analysis showed an enrichment of genes involved in brain development (Bonferroni adjusted *p*-value 8.29e-09) and morphogenesis (adjusted *p*-value 2.67e-07); many homeobox genes belong to this later set of genes reflecting probably the differences in tumor location.

**Fig. 1:**
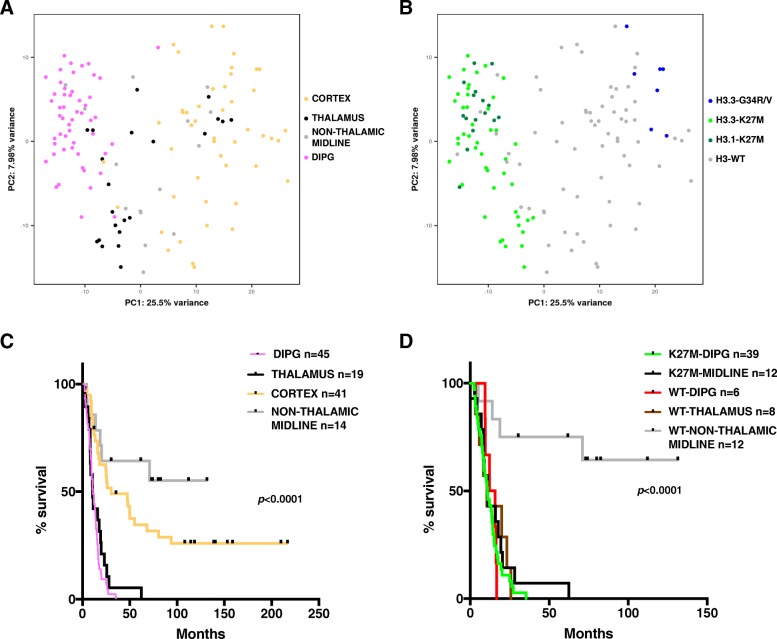
Gene-expression based classification of high-grade gliomas and corresponding survival analyses. Principal component analysis of microarray GE profiling of 119 high-grade gliomas. In total, 131 data points are represented as 11 samples were duplicated allowing to monitor the batch effect correction. The genes associated with the highest standard deviation were selected (*n* = 120 genes) for the analysis and the tumors were color-coded according to their location (**a**) or mutational histone H3 status (**b**). Four groups were defined in the upper left panel corresponding to cortical (yellow), thalamic (black), pontine (pink) and non-thalamic midline (grey) glioma. In the right panel, the samples were divided in 4 subgroups according to the mutational status of histone H3 genes: H3.3-G34R (blue), H3.3-K27M (light green), H3.1-K27M (dark green) mutated tumors and tumors without any alteration of either *H3F3A*, *HIST1H3B* and *HIST2H3A* genes (grey). **c** Kaplan–Meier of the overall survival of patients with a high-grade glioma stratified by their location. DIPG (green) and thalamic (black) tumors are associated with the shortest overall survival (median of 11.1 months and 10.8 months respectively). The midline tumors (grey) which are located outside the thalamus show the most favorable prognosis. The subgroup of cortical tumors (yellow) shows an intermediate phenotype (median survival 30.5 months). Log rank test *p-*value < 0.0001. **d** Kaplan–Meier survival curves of patients with a midline HGG stratified by both tumor location and H3-K27 mutational status. The overall survival is rather similar for all tumor subgroups (overall median survival about 10.8, 13.86, 10.02, 10.5 months for K27M DIPG, WT DIPG, K27M midline, WT thalamus, respectively) except for the WT non-thalamic midline tumors presenting a much better prognosis. Log rank test *p-*value < 0.0001

Principal component analysis (PCA) was performed to highlight the principal sources of variation among pHGG tumors (Additional file 2: Table S2: weight in principal components 1 and 2 associated to each gene). The DIPG group appeared rather homogenous as all samples clustered together in the PCA plot, separated from the tumors originating from thalamic or cortical areas of the brain which were more scattered (Fig. 1a). Considering the mutational status of histone H3 genes, the results clearly showed that H3-K27M tumors, whichever their pontine or thalamic location, could be separated from the wild-type and G34R/V tumors on the first principal component (Fig. 1b). Indeed, all H3-K27M mutated thalamic and spinal tumors were close to DIPG samples, whereas histone H3 wild-type thalamic tumors are distributed on the right side of the plot among histone H3 wild-type non-thalamic midline and cortical tumors. Interestingly, 5 DIPG without any mutation in *H3F3A*, *HIST1H3B/C* and *HIST2H3A/C* were located within the K27M DIPG subgroup. These tumors all showed H3K27-trimethylation loss by immunohistochemistry (Additional file 3: Figure S1A)*.* Unsupervised K-means analysis was performed on the same dataset (using *k* = 2 which showed the best BIC value) and led to a similar conclusion as the k-mean group 1 corresponded to H3-K27M and H3-wild type samples presenting H3K27-trimethylation loss whereas k-mean group 2 contained all other pHGG tumors (Additional file 3: Figure S1B). Additionally, this GE dataset was analyzed in parallel with another dimension reduction and visualization technique for high-dimensional data, t-SNE, as it was shown to be more robust than PCA with respect to outliers. Moreover, t-SNE has also been frequently used for pediatric brain tumor classification based on DNA methylation profiles [23]. This analysis of GE profiles led to similar observations, re-iterating the similarity of H3-K27M thalamic midline and DIPG (Additional file 3: Figure S1C-D). Histone H3-G34R/V samples were tightly clustered together in particular in this analysis, thus reflecting a strong similarity in their gene expression profiles.

In agreement with these observations, a huge number of differentially expressed genes were identified between H3-K27M and H3-wild-type tumors as well as between H3-K27M and H3-G34R tumors (adjusted *p*-value < 0.01) in comparison with the other contrasts (Additional file 3: Figure S1E and Additional file 4: Table S3). In contrast, only 14 genes were significantly modulated between H3-G34R and H3-WT tumors which were not discriminated by PC1 (Fig. 1b). Gene ontology analysis showed an important enrichment of modulated genes associated with neurogenesis (4.37e-12 – 1.41e-11) and neuron differentiation (3e-07 - 4.87e-13) signaling pathways in both contrasts (H3-K27M *vs.* H3-WT and H3-K27M *vs.* H3-G34R) , as well as an upregulation of genes involved in ion transmembrane transport (1.6e-04) and apoptotic processes (5.9e-18) and an downregulation of genes linked to cell cycle (8.3e-11) and gliogenesis (6.87e-10) in the case of the comparison between H3-wild-type and H3-K27M tumors. Also, geneset enrichment analysis (GSEA) identified the ontology GO_oligodendrocyte_differentiation (Enrichment Score 0.70) enriched in upregulated genes in H3-K27M tumors and GO_Cerebral_cortex_neuron_differentiation (Enrichment Score − 0.79) enriched in genes upregulated in H3-G34R. Some of these biological processes were previously identified as significantly enriched in differentially expressed genes when comparing H3.1- and H3.3-K27M tumors [2].

We next conducted a survival analysis on this cohort (*n* = 119) using only location information and then both location and H3 mutation status for patient stratification (Fig. 1c and d). DIPG and thalamic tumors were associated with similar poor prognosis, i.e. 11.1 and 10.8 months median OS, respectively. Non-thalamic midline tumors exhibited the best prognosis (median OS not reached), whereas tumors arising in the cortex presented an intermediate outcome with a median survival around 30.5 months (*p-*value < 0.0001, Fig. 1c). Focusing on Kaplan-Meier estimates for midline tumors, our data clearly indicate that H3-WT non-thalamic midline have a significantly higher overall survival, whereas the other midline malignant gliomas (mostly thalamic), with or without alteration of histone H3 genes, display equivalent poor survival (*p-*value < 0.0001, Fig. 1d).

### Methylation profiling separates *HIST1H3B* and *H3F3A* K27M tumors

**Fig. 2:**
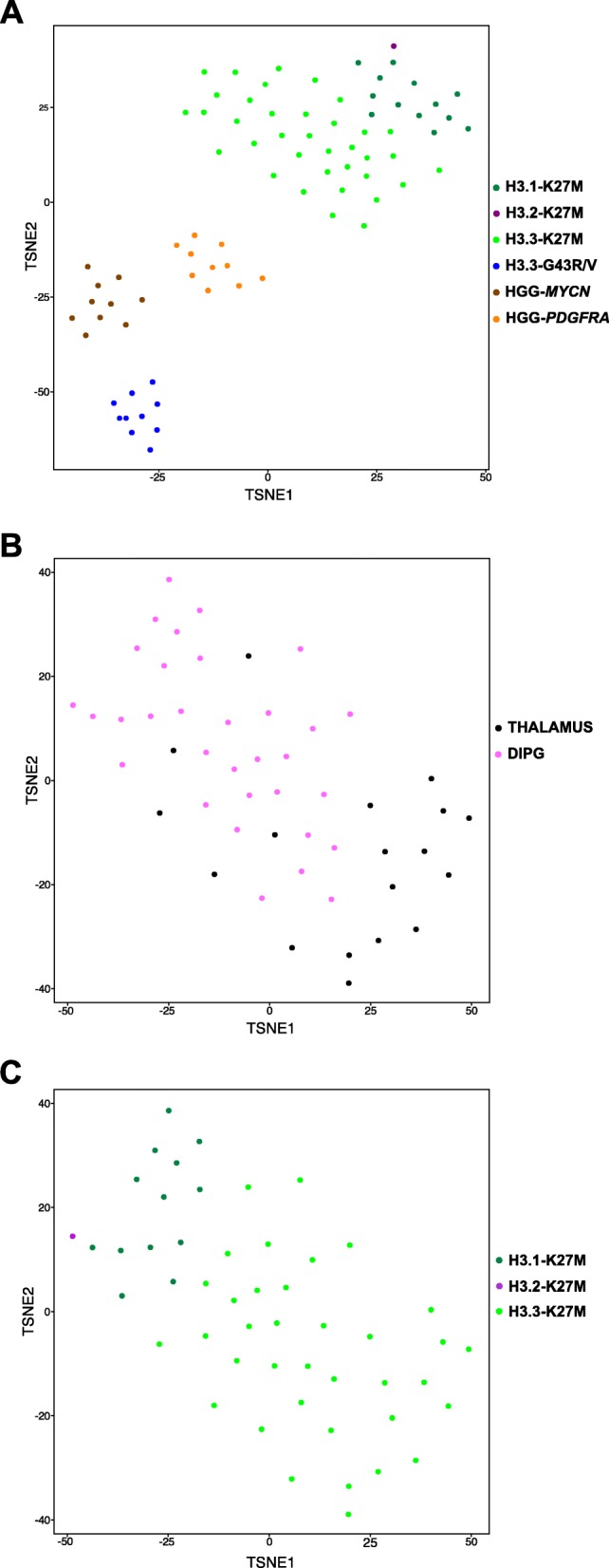
Classification of high-grade gliomas based on genome-wide DNA methylation profiles. **a** t-SNE analysis of the methylation profiles of 80 pediatric high-grade gliomas using the topmost differentially methylated probes across the sample set (s.d. > 0.25). Midline tumors are color-coded according to the histone H3 gene mutated: dark green for H3.1-K27M (*n* = 13), purple for H3.2-K27M (*n* = 1) and light green for H3.3-K27M tumors (*n* = 36). Others H3-WT high-grade glioma are also presented: H3.3-G34R mutated tumors (*n* = 10, blue), *PDGFRA* (*n* = 10, orange) and *MYCN* (*n* = 10, brown) amplified tumors. **b**-**c** Analysis of methylation patterns of 50 pediatric H3-K27M midline tumors by t-SNE indicates that H3.1-K27M and H3.3-K27M tumors are clearly distinct from each other. Dimensionality reduction and visualization of methylome data was performed by t-SNE after selection of the probes with the greatest variance (*n* = 10,000; See Methods). Samples were color-coded according to their location (**b**), the histone H3 gene mutated (**c**). t-SNE show two main clusters corresponding to H3.1/H3.2-K27M and H3.3-K27M subgrouping

Previous studies have shown that genome-wide DNA methylation data can provide a robust classification of pediatric brain tumors into clinically meaningful epigenetic subgroups mostly characterized by recurrent genetic alterations [14, 15, 22]. Consequently, we compared the methylation profiles of K27M-mutated diffuse midline gliomas (including DIPG) to G34R-mutated tumors and well-characterized supratentorial tumors without mutation in histone H3 genes, i.e. *MYCN* and *PDGFRA* tumor subgroups (Fig. 2a, Table 2) [15]. Eighty primary tumor samples were used in this analysis and t-SNE visualization of the DNA methylation data was conducted. We confirmed that H3-G34R, *PDGFRA* and *MYCN* subgroups constitute 3 distinct homogenous entities, as they defined three distinct clusters. All H3-K27M samples were located on the opposite side of the 2D representation, reflecting important differences in the methylome compared to these three well-defined pHGG subgroups. This observation was thus concordant with our results on GE profiling by microarray.

In addition, the same methylation profiling splits H3-K27M samples in two subgroups that corresponded to either H3.1 or H3.3 mutated tumors. The obvious separation of these tumors in an analysis containing other very distinct biological entities clearly indicated the significant difference between them. Also, the unique H3.2-K27M sample appeared closer to H3.1-K27M than H3.3-K27M samples (Fig. 2a).

The same classification by t-SNE was repeated for the subset of H3-K27M mutated midline gliomas. First, t-SNE analysis did not reveal a segregation of these samples according to their location, as all DIPG and thalamic midline were scattered in the 2D plot (Fig. 2b). Conversely, when considering samples based on the mutated histone H3 gene, the t-SNE analysis clearly highlighted two non-overlapping subgroups corresponding to H3.1/H3.2-K27M and H3.3-K27M classes (Fig. 2c). This observation indicates that H3.3-K27M DIPG are closer to other midline H3.3-K27M HGG than to H3.1-K27M DIPG. The histone H3 variant affected by the K27M substitution thus has a stronger correlation with the modulation of DNA methylation profile than the tumor location across the midline.

**Fig. 3:**
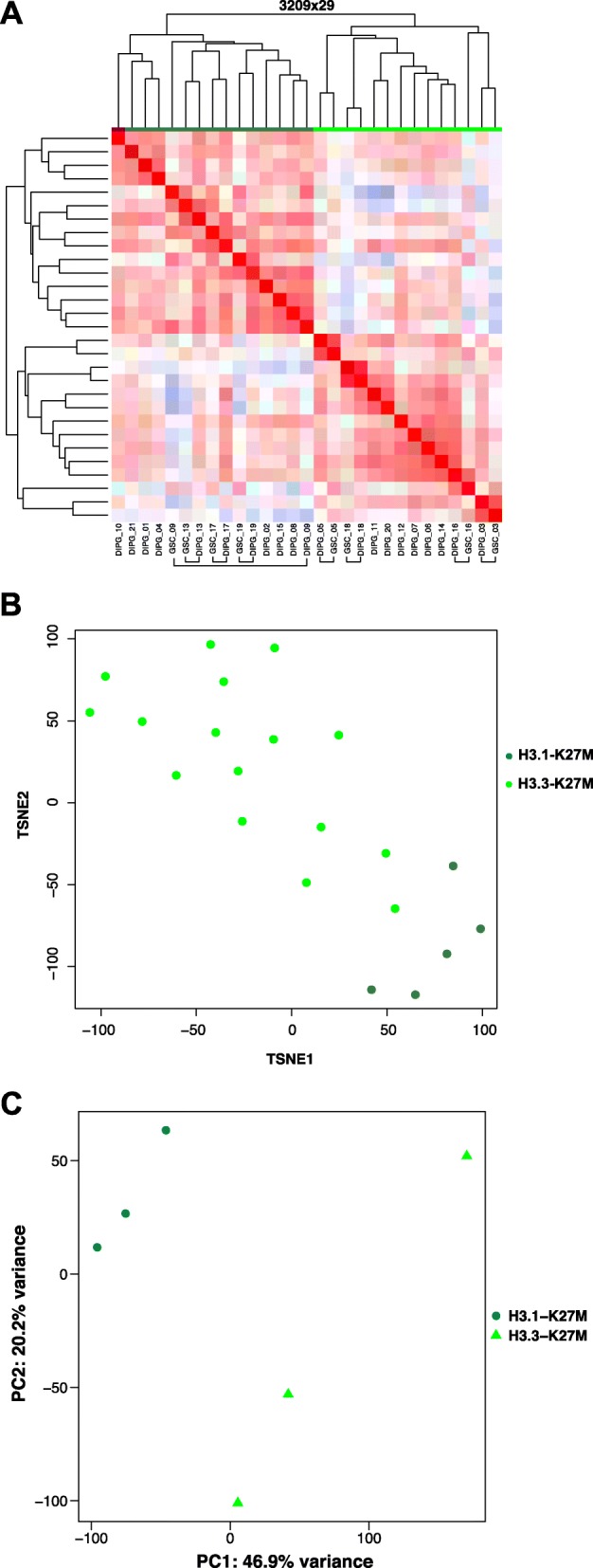
Subclassification of DIPG based on RNA-seq, methylome and H3K27me3 epigenetic profiles. **a** Heatmap and hierarchical clustering of the Pearson correlation matrix of 19 DIPG and 8 matched GSC cultures (H3.3-K27M and H3.1-K27M in light and dark green respectively) across 3209 probes used for DNA methylation measurements. **b** t-SNE analysis of RNA-seq data in DIPG. RNA sequencing of 21 DIPG samples was performed and the genes associated with the highest standard deviation (*n* = 250) were selected to conduct t-SNE analysis. The tumors were color-coded according to histone H3 mutated, i.e. H3.3-K27M in light green and H3.1-K27M in dark green. **c** Principal component analysis of 3 H3.1- and 3 H3.3-K27M GSC models of DIPG based on H3K27me3 epigenetic mark profiling (*n* = 16,977 genomic intervals)

An additional analysis was performed on a subset of 21 primary DIPG tumors and 8 glioma stem-like cells (GSCs) deriving from these same biopsies. The sample classification by unsupervised hierarchical clustering confirmed the previous result, with two main clusters corresponding to H3.3-K27M and H3.1/2-K27M samples (Fig. 3a). Additionally, we observed that the majority of the GSCs clustered with their corresponding primary tumors indicating the close similarity of their methylome profile. This consequently underlined that GSC population remained very similar to their primary counterpart with respect to DNA methylation, reflecting the variation in DNA methylation observed between the two subgroups of H3.1 and H3.3 mutated tumors.

### RNA-seq profiling also discriminates *HIST1H3B/C* and *H3F3A* K27M mutated gliomas

DNA methylation is a relatively stable component of the epigenome involved in the establishment and maintenance of distinct gene expression patterns. Consequently, we decided to evaluate if the different DNA methylation profiles were associated with distinct transcriptome profiles. PCA analysis of GE measurements by microarrays did not clearly discriminate H3.1 and H3.3 mutated tumor samples (Fig. 1b). Indeed, even if H3.1-K27M DIPG were closer to each other in the 2-dimensional PCA plot, they are surrounded by H3.3-K27M samples. However, as microarray data were generated in several batches, we could not exclude that this could obscure the dataset, despite the use of a batch correction method. Therefore, we took advantage of a RNA-seq study of 21 new H3-K27M DIPGs samples which appeared more suitable as it provides an exhaustive measurement of transcriptome in contrast to microarray analysis. Grouping of tumors based on their RNA-seq expression profiles in either t-SNE or PCA classifications confirmed the discrimination of H3.1-K27M from H3.3-K27M tumors observed in our DNA methylation study (Fig. 3b and Additional file 5: Figure S2).

### H3.1- and H3.3-K27M mutations are associated with different genomic distribution of the H3K27me3 epigenetic mark

To complement the description of the epigenetic landscape of DIPGs, we decided to assess the direct epigenetic consequences of the K27M mutation in H3.1-K27M and H3.3-K27M tumors, i.e. the loss and redistribution of the trimethylation mark at position K27. We thus took advantage of 6 GSC models of different genotypes as an expandable source of material and interrogated the genome-wide distribution of this mark with ChIP-seq. We conducted a PCA analysis on all genomic regions showing enrichment for this mark and could again separate the samples based on the type of mutated histone (Fig. 3c). This implied that despite the similar biochemical impact of both H3.1-K27M and H3.3-K27M mutated histones on PRC2, interferences of the two different mutated histones H3 on PRC2 is not similarly distributed in the genome and this may induce meaningful changes in the epigenetic landscapes of these DIPGs.

**Fig. 4:**
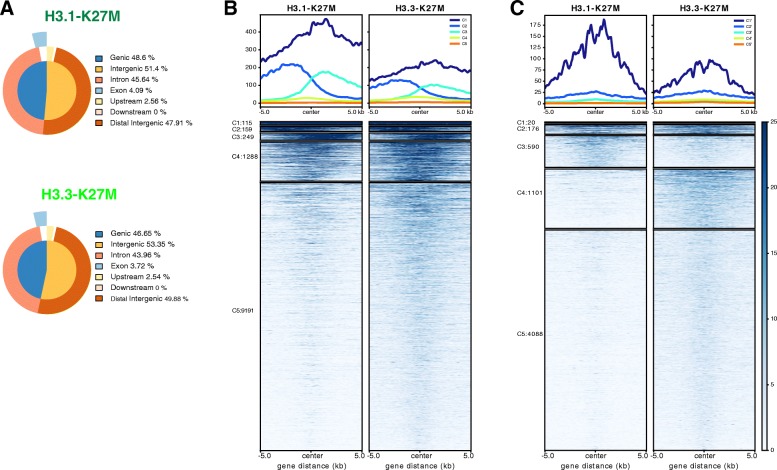
Distribution of H3K27me3 epigenetic marks in in vitro models of DIPG. **a** Genome distribution of H3K27me3 peaks in H3.1- and H3.3-K27M GSC cells. Pie-charts represent the genomic annotation of the genomic loci bound by H3.3K27me3 in each subgroup performed using ChIPseeker package. The majority of the H3K27me3 occupied regions are located within the distal intergenic regions, and a small number of peaks are located in upstream regions. The percentage of each feature in H3.1- and H3.3-K27M subgroups is indicated in the legend. **b**-**c** H3K27me3 levels in overlapping (**b**) or non-overlapping (**c**) gene regions of 10 kb were normalized to equivalent total number of tags in the samples (in columns), and genomic intervals were subsequently clustered by k-means (*k* = 5). Left and right borders represent − 5 kb and + 5 kb, respectively. Blue color scale bar indicates relative coverage. Average signal of all H3K27me3 peak regions of each cluster is presented at the top. The number of genomic loci in each cluster is indicated in the legend

The average signal of all H3K27me3 peak regions was equivalent in H3.1- and H3.3-K27M tumors (Additional file 6: Figure S3A, top). We plotted the ChIP-seq signal of the union of H3K27me3 peaks identified from all the GSC samples and did not observed a specificity of the genomic regions presenting a H3K27me3 deposition between H3.1 and H3.3 K27M subgroups but rather a difference in the level of the signal enrichment (Additional file 6: Figure S3A, bottom). However, the genome distribution of H3K27me3 showed an enrichment of peaks in distal intergenic regions in H3.3-K27M GSC and in contrast in intronic and at a lesser extent exonic regions in H3.1-K27M cells (Fig. 4a).

Subsequently, we divided the active genomic loci presenting H3K27me3 deposition according to their location. Overall, the global signal was significantly higher in regions overlapping genes *versus* intergenic regions (Additional file 6: Figure S3B-C). The heatmap displayed that the majority of regions overlapping genes presented a lower level of H3K27me3 in H3.1-K27M samples (Additional file 6: Figure S3C). This observation was concordant with a higher number of expressed genes in H3.1, which are moreover associated with higher expression levels (Additional file 6: Figure S3D).

The clustering of genomic intervals by k-means highlighted the existence of distinct subgroups of loci, smaller gene clusters with a significantly higher level of H3K27me3 in H3.1-mutated cells (C1, C2 and C3 for genic and C1’ and C3’ for intergenic loci, Fig. 4b and c respectively). Consequently, the vast majority of loci were enriched in K27me3 in H3.3-mutated cells.

**Fig. 5:**
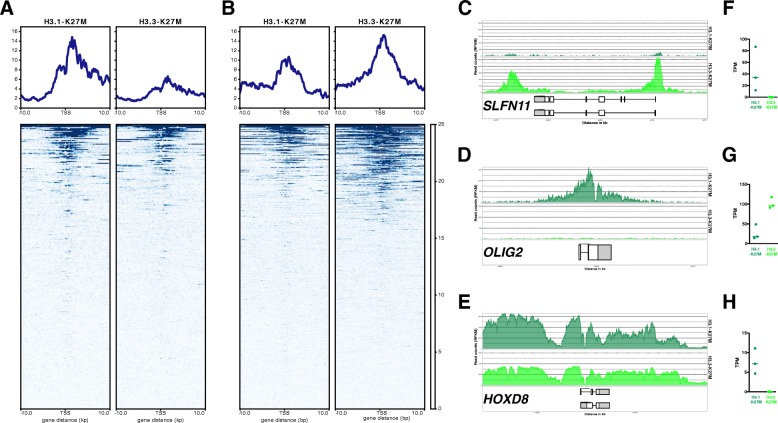
**a**-**b** H3K27me3 ChIP-seq signal at promoter regions of upregulated (**a**) and downregulated (**b**) genes between H3.1- and H3.3-K27M GSCs (adjusted *p*-value < 0.01). The average occupancy is centered on TSS and extended 10 kb upstream and downstream (− 10 kb and + 10 kb, respectively). Blue color scale bar indicates relative coverage. **c**-**e** H3K27me3 levels found at the loci of selected genes showing increased (*OLIG2* and *HOXD8*) or decreased (*SLFN11*) mark deposition in H3.1-K27M. Read coverage around the genes of interest is represented in RPKM and gene structure from Ensembl database is shown below. **f**-**h** Expression level in tpm of *OLIG2*, *SLFN11* and *HOXD8* measured by RNA-seq in GSCs

Thereafter, we focused on the differentially expressed genes identified by RNA-seq between the 2 subgroups (adj *p*-value< 0.01). The results showed that upregulated genes in H3.1-K27M are associated with a lower average signal of H3K27me3 and *vice et versa* for downregulated genes (Fig. 5a). The ChIP-seq read coverage are shown for two representative genes, *OLIG2* and *SLFN11,* even if distinct localization of H3K27me3 is observed between both, located mainly upstream and downstream of transcribed region for *SLFN11* and overlapping gene body for *OLIG2* (Fig. 5b and c). However, gene expression modulations between H3.1- and H3.3-mutated tumors was not restricted to changes in H3K27me3 deposition at the close vicinity of the gene body as shown for *HOXD8* which is upregulated and associated with an increased H3K27me3 deposition in H3.1-K27M tumors (Fig. 5d).

## Discussion and conclusions

The recent update of the WHO classification aggregated DIPG and infiltrating glial neoplasms of the midline presenting a H3-K27M substitution as a new entity: diffuse midline glioma (DMG) H3 K27M-mutant. But this implied that the histone H3-K27M would be a stronger driver of oncogenesis than location. We used a pHGG cohort at diagnosis to evaluate the similarity of these H3-K27M mutated tumors at both DNA methylation and gene expression levels and compared them to other pHGG tumor subgroups in order to support or question the new update of the WHO classification [23].

Tumor classification based on microarray gene expression profiling revealed that K27M mutated tumors, either thalamic or pontine, can be discriminated from all others. Consequently, the molecular subtype appears to influence more the gene expression profile than the infratentorial *vs.* supratentorial location of the tumor in the brain. Alternatively, location may not be considered at the structural level (i.e. brainstem *vs.* thalamus) but rather at the embryological level (midline *vs.* hemispheres) thus unifying midline tumors. Accordingly, survival analyses highlighted a similarly poor prognosis for DIPG and thalamic tumors, either mutated or not for histone H3. The bad outcome of all midline gliomas with K27M mutations was also observed by Karreman et al. [13]*.* Taken together these data support the rationale to define the same treatment paradigms for both midline K27M tumors and DIPG.

The stratification based on DNA methylation profiling of our pHGG population also supports the similarity between thalamic and pontine H3-K27M tumors. Our results are concordant with previous reports concerning the discrimination of G34R/V and K27M mutated tumors depending on DNA methylation [18, 23]. Moreover, t-SNE analysis highlighted a clear distinction of H3-K27M tumors from all other pHGG subtypes. Indeed, G34 mutated tumors, *PDGFRA* and *MYCN* subtypes represent three homogenous groups distinct from K27M tumors.

The DIPG median survival was similar to the large retrospective pHGG cohort recently analyzed by MacKay and collaborators [18]. However, midline and hemispheric tumors were associated with longer median survival in our cohort, 18 *versus* 13.5 months and 30.5 *versus* 18 months, respectively. Survival analyses also pointed out a significantly better outcome of histone H3 wild-type non-thalamic midline tumors, which likely reflects that they may be less diffusely growing gliomas and could therefore be more amenable to surgical resection, or that they exhibit a behavior of low-grade gliomas.

Finally, in the gene expression analysis some diffuse midline gliomas without any H3-K27M mutation are grouped with the H3K27M tumors. Interestingly, they all exhibit a loss of the H3K27me3 mark as well. Thus, defining the entity by the H3K27M mutation only may therefore be too restrictive. Further studies are needed to sort this issue, especially since diffuse pontine and thalamic malignant gliomas have a poor prognosis irrespective of the presence of an H3K27M mutation or not as also recently shown in the HERBY trial (Mackay et al., Cancer Cell 2018).

Interestingly, our methylation profiling data showed a subclassification of DMG, H3 K27M-mutant into two subgroups according to the histone gene affected by the K27M substitution, i.e. *H3F3A* or *HIST1H3B/C*. The sole H3.2-K27M sample clustered together with H3.1-K27M tumors, as expected given that they are both canonical histone H3 with identical role in the cell [24]. Yet, the similarity of H3.1 and H3.2 mutated tumors should be confirmed with additional H3.2 mutated samples from other cohorts, as only two were reported in the literature [2, 18]. Histone H3.1 and H3.3-K27M tumors were also discriminated by RNAseq transcriptome profiling, supporting their intrinsic divergence. This could support the recently reported superiority of RNAseq over expression microarrays for tumor classification purposes [28]. MacKay *and coll*. did not report this distinction between H3.1 and H3.3 mutated tumors using DNA methylation profiling. This difference might result from a 10 times smaller proportion of H3.1 mutated samples analyzed (8 out of 441 samples) hiding out the variability brought by these tumors in their huge dataset. In addition, we used a 7 times larger set of probes (10,000 instead of 1381) that might have captured more variations in the overall pHGG DNA methylation landscape.

It is assumed -and was recently demonstrated by Hoadley et al., that DNA methylation can reflect the epigenetic memory of cancer cell-of-origin [11]. Indeed, DNA methylation is inherited through successive division and is shown to be not only tumor-type specific, but can also reflect the cell type and differentiation state of the transformed cells [6]. The clear separation by DNA methylation profiling of H3.1-K27M from H3.3-K27M tumors may support that these tumors would arise from distinct cells of origin or at distinct differentiation steps in the lineage. This strongly corroborates our previous results showing that DIPG can be divided in two main H3.1-K27M and H3.3-K27M tumor subgroups, associated with distinct histological and molecular phenotypes, age of onset and location along the midline, H3.1-K27M mutation being almost exclusively seen in the brainstem while H3.3-K27M mutation are distributed everywhere along the midline [2]. Also, the conservation of DNA methylation discrepancies in GSCs confirm they are intrinsic characteristic of the tumor cells as opposed to the peri-tumor stroma. Furthermore, we demonstrate that despite the same global biochemical consequence of the H3K27M driver mutation, significant differences exist in the H3K27me3 landscape relying on the type of histone H3 variant affected (i.e. H3.1 or H3.3) as shown by PCA.

As a whole, the distribution of the H3K27me3 marks along the genome is different, both at the quantitative and qualitative levels. Average level of trimethylation at K27 is similar in both subtypes since only a small number of loci are highly enriched in H3K27me3 in H3.1 K27M mutated tumors, whereas the majority of the regions presenting this epigenetic mark are associated with a higher signal in H3.3-K27M. These H3K27me3 variations among the two subgroups are associated with the modulations of gene expression, many more genes being repressed in H3.3-K27M tumors. Qualitatively, K-means clustering of the distribution of this mark identified 5 clusters of genic regions and 5 clusters of intergenic regions differentially trimethylated at position K27 in the two subgroups of DIPG. We show that among differentially expressed genes, levels of H3K27me3 are anti-correlated with gene expression in general. However, gene expression could not be strictly explained by the levels of H3K27me3 in all cases leaving the possibility of additional levels of regulation for gene expression in DIPG.

Overall, we provide molecular and clinical evidence in favor of the unification of all midline K27M mutated tumors that was proposed in the 2016 WHO CNS classification based on their common driver mutation. As such, these gliomas need to be considered as a unique entity in future clinical trials. Further analyses on the biology of H3.3 and H3.1 mutated diffuse gliomas are required to explain the distinction we have reported so far; this could allow testing specific precision medicine approaches in these two subgroups of diffuse midline gliomas.

## Additional files

Additional file 1: **Table S1.** Summary of the 215 pHGG samples analyzed by either gene expression microarray, RNA-seq or methylation array. (XLSX 9 kb)

Additional file 2: **Table S2.** PCA weights in gene expression analysis of pHGG. Table containing the weights of the 2 first principal components of each of the 120 genes used for the analysis of gene expression of the pHGG by PCA (Fig. 1). (XLSX 14 kb)

Additional file 3: **Figure S1.** A. Principal component analysis of microarray GE profiling of 119 high grade gliomas as presented in Fig. 1b colored by mutational histone H3 status. Five pontine WT tumors that also harbor a H3K27 trimethylation loss by IHC are highlighted with red arrowheads. B- The gene expression data of the 120 genes associated with the highest standard deviation (*n* = 120 genes) were used for k-means analysis (*k* = 2) and the results represented in the same PCA as in panel A. The samples were color-coded according to k-means results, symbols reflect the histone H3 mutational status and their location are indicated in the plot. C-D. t-SNE analysis of the GE profiles of 119 pediatric high-grade gliomas using the genes associated with the highest standard deviation (*n* = 120 genes). The tumors were color-coded according to their location (A) or mutational histone H3 status (B) as described in Fig. 1. E- Overlapping of the gene lists resulting from differential analysis between: H3-K27M and wild-type tumors, H3-K27M and G34R tumors, H3-G34R and wild-type tumors, H3.1 K27M and H3.3-K27M tumors (adjusted *p*-value< 0.01). (PDF 224 kb)

Additional file 4: **Table S3.** Lists of differentially expressed genes in microarrays data from H3-K27M *vs.* H3-WT, H3-G34R vs. H3-K27M, H3-G34R *vs.* H3-WT comparisons (adj *p*-value< 0.01). (XLSX 174 kb)

Additional file 5: **Figure S2.** Principal component analysis of the GE profile of 21 DIPG using the gene associated with the highest standard deviation (*n* = 250 genes, H3.1-K27M DIPG in dark green and H3.3-K27M DIPG in light green). (PDF 54 kb)

Additional file 6: **Figure S3.** A-C. Metaplots showing average signal accumulation in reads of all the regions bound by H3K27me3 in at least one sample (A, *n* = 16,979) or H3K27me3 occupied regions with or without overlapping genes (B, *n* = 11,003 and C, *n* = 5976 respectively) in both H3.1- and H3.3-K27M GSC cells. Each plot is centered on the summit of the average occupancy and extended 10 kb upstream and downstream (− 10 kb and + 10 kb, respectively). Below the metaplots, heatmaps illustrating average H3K27me3 levels in the 20 kb genomic intervals centered on the summit of the peak in each subgroup are presented. D. Violin plot displaying transcript expression level of RNA-seq data in tpm in H3.1- and H3.3-K27M subgroups. Boxplots represent the 5th,25th,75th and 95th percentiles and the median of the transcript distribution, The distributions were divided in 4 categories: non expressed genes (< 0.1 tpm), low expressed genes (from 0.1 to 1 tpm), intermediate (from 1 to 10 tpm) and highly expressed genes (> 10 tpm). (PDF 2819 kb)
