# Supplementary figures and images for: Transcriptomic and epigenetic profiling of ‘diffuse midline gliomas, H3 K27M-mutant’ discriminate two subgroups based on the type of histone H3 mutated and not supratentorial or infratentorial location
Source: Acta Neuropathol Commun. 2018 Nov 5;6:117. doi: 10.1186/s40478-018-0614-1 (PMC6219253; doi:10.1186/s40478-018-0614-1)

A

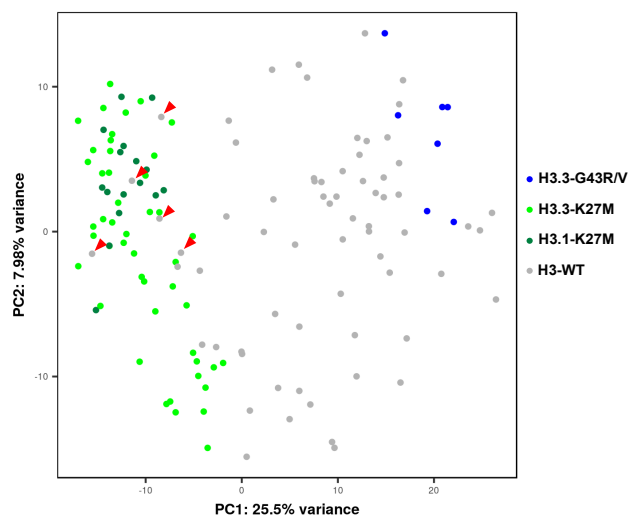

B

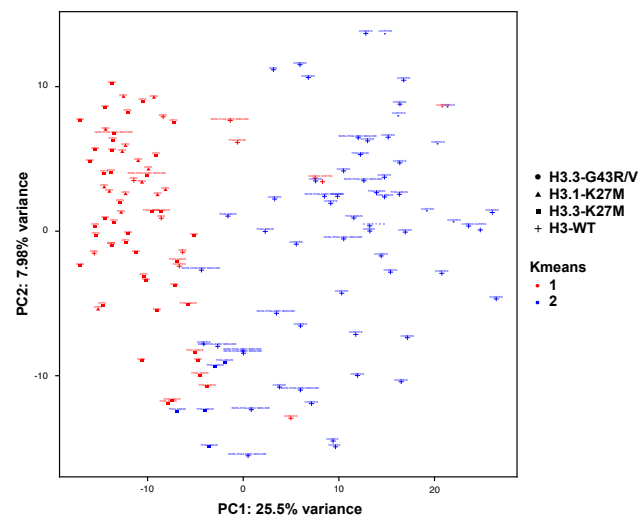

C

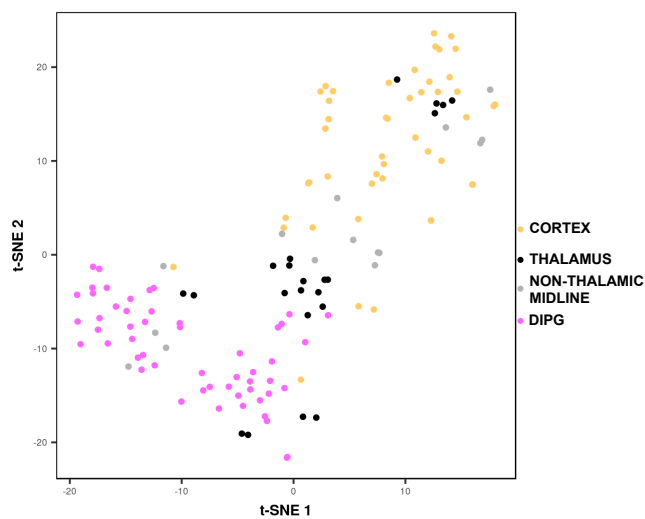

D

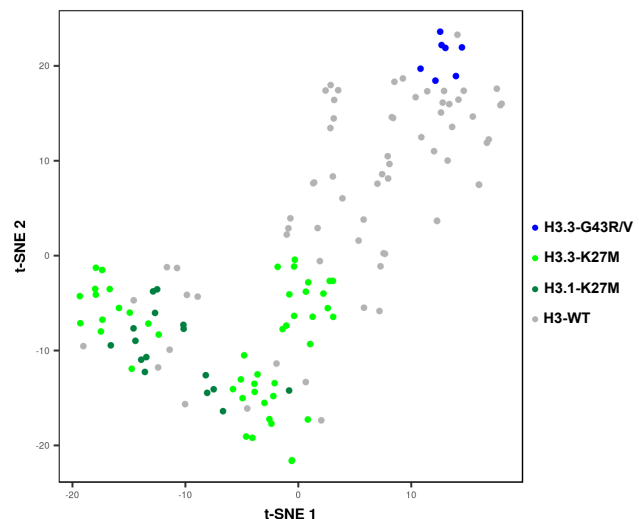

E

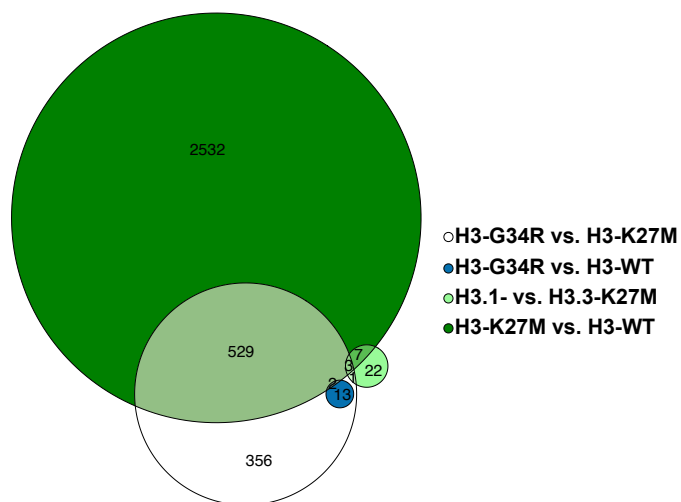

Supplement: Supplementary file 3 — Figure S1. A. Principal component analysis of microarray GE profiling of 119 high grade gliomas as presented in Fig. 1b colored by mutational histone H3 status. Five pontine WT tumors that also harbor a H3K27 trimethylation loss by IHC are highlighted with red arrowheads. B- The gene expression data of the 120 genes associated with the highest standard deviation (n = 120 genes) were used for k-means analysis (k = 2) and the results represented in the same PCA as in panel A. The samples were color-coded according to k-means results, symbols reflect the histone H3 mutational status and their location are indicated in the plot. C-D. t-SNE analysis of the GE profiles of 119 pediatric high-grade gliomas using the genes associated with the highest standard deviation (n = 120 genes). The tumors were color-coded according to their location (A) or mutational histone H3 status (B) as described in Fig. 1. E- Overlapping of the gene lists resulting from differential analysis between: H3-K27M and wild-type tumors, H3-K27M and G34R tumors, H3-G34R and wild-type tumors, H3.1 K27M and H3.3-K27M tumors (adjusted p-value< 0.01). (PDF 224 kb) [file 40478_2018_614_MOESM3_ESM.pdf]

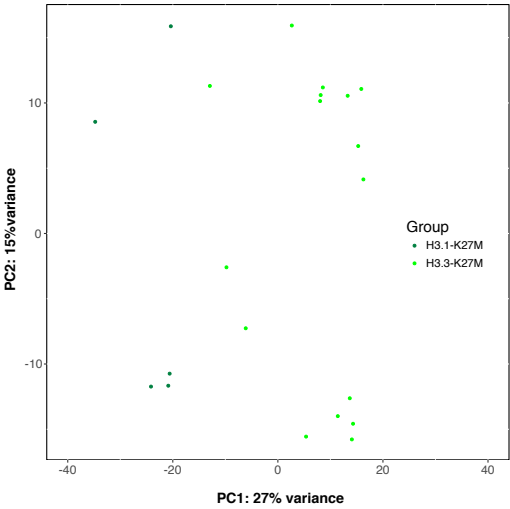

Supplement: Supplementary file 5 — Figure S2. Principal component analysis of the GE profile of 21 DIPG using the gene associated with the highest standard deviation (n = 250 genes, H3.1-K27M DIPG in dark green and H3.3-K27M DIPG in light green). (PDF 54 kb) [file 40478_2018_614_MOESM5_ESM.pdf]

Figure S3. Castel *et al.*

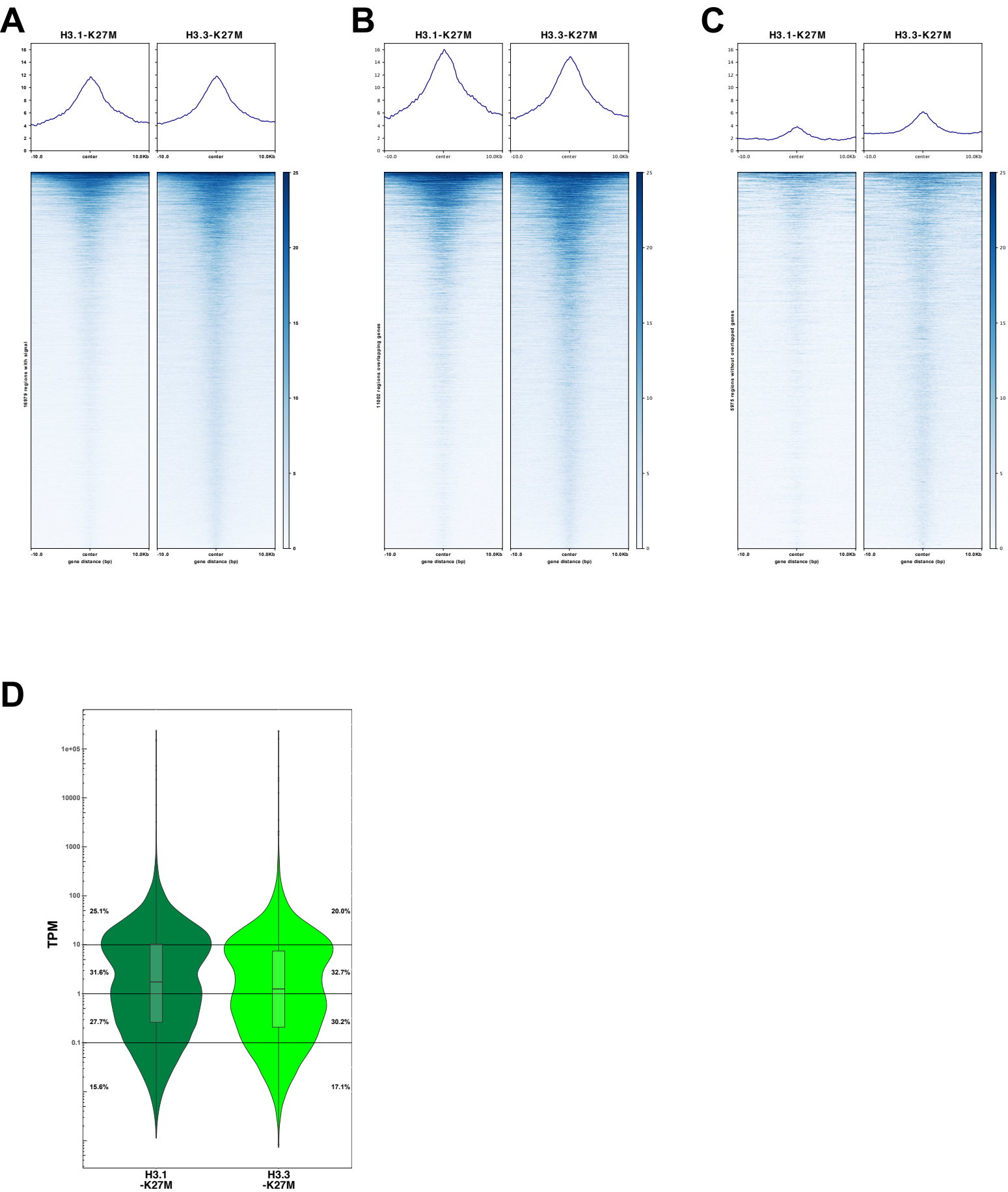

Supplement: Supplementary file 6 — Figure S3. A-C. Metaplots showing average signal accumulation in reads of all the regions bound by H3K27me3 in at least one sample (A, n = 16,979) or H3K27me3 occupied regions with or without overlapping genes (B, n = 11,003 and C, n = 5976 respectively) in both H3.1- and H3.3-K27M GSC cells. Each plot is centered on the summit of the average occupancy and extended 10 kb upstream and downstream (− 10 kb and + 10 kb, respectively). Below the metaplots, heatmaps illustrating average H3K27me3 levels in the 20 kb genomic intervals centered on the summit of the peak in each subgroup are presented. D. Violin plot displaying transcript expression level of RNA-seq data in tpm in H3.1- and H3.3-K27M subgroups. Boxplots represent the 5th,25th,75th and 95th percentiles and the median of the transcript distribution, The distributions were divided in 4 categories: non expressed genes (< 0.1 tpm), low expressed genes (from 0.1 to 1 tpm), intermediate (from 1 to 10 tpm) and highly expressed genes (> 10 tpm). (PDF 2819 kb) [file 40478_2018_614_MOESM6_ESM.pdf]
